# Treatment of Wastewaters with Zirconium Phosphate Based Materials: A Review on Efficient Systems for the Removal of Heavy Metal and Dye Water Pollutants

**DOI:** 10.3390/molecules26082392

**Published:** 2021-04-20

**Authors:** Monica Pica

**Affiliations:** Department of Pharmaceutical Sciences, University of Perugia, Vial del Liceo 1, 06123 Perugia, Italy; monica.pica@unipg.it

**Keywords:** zirconium phosphate, wastewater pollutants, ion exchange, heterogeneous photocatalysis

## Abstract

Layered zirconium phosphate (ZrP) is a versatile material with phosphate (POH ) groups able to exchange inorganic and organic cations or to intercalate basic molecules. The present review deals with the use of this material as a sorbent for heavy metal cations or dye molecules in wastewater treatments. The possibility to combine ZrP with polymers or other inorganic materials, in order to have suitable systems for real and large scale applications, was investigated, as well as the combination with photocatalytic materials to obtain hetrogeneous photocatalysts for the capture and photodegradation of organic dye molecules.

## 1. Introduction

The progress of society led, on one hand, to a significant improvement in the quality of people’s life, but, on the other hand, to the significant anthropogenic pollution of soil, water and air due to the intensive exploitation of natural resources. The scientific community is called to support society in this challenge, finding realistic and effective strategies to control, reduce and remedy environmental pollution. According to The Longman Dictionary of Environmental Science, environmental pollution is defined as “any harmful or undesirable change in the physical, chemical or biological quality of air, water or soil” [1].

The present review is focused on water pollution and, specifically, on the strategies to remove some categories of common pollutants from water.

Freshwater on Earth is only 3% of the total water, and about 20% of the population live in conditions of a lack of freshwater. According to the World Health Organization (WHO), more than one million people consume non-potable water, with letal effects causing about 30,000 daily deaths [2].

Inorganic compounds such as phosphates and nitrates, dyes and phenolic compounds, as well as pesticides, recalcitrant organic matter, sediments, heavy metal ions, and products derived from pharmaceutical preparations and industrial activities (plastic, leather, textile, paper, ceramics, glass, cosmetics, food, paints, soap, wax, biomedicine industry) are the most common water pollutants, which are often found in water resources as mixtures, leading to dangerous synergistic effects and complicating their detection, quantification and removal [2,3].

Dyes and heavy metal ions are among the main water pollutants derived from industrial activities. Synthetic dyes find application in paper printing, photography, and the pharmaceutical, cosmetic, food and textile industries, and their history started in 1856 when the first aniline dye, Mauveine, was discovered by William Henry Perkin. Dyes possess unsaturated groups, with conjugated chemical bonds, and often complex structures which are responsible for light absorption and emission in the visible region and for the difficulty of removal from water [2].

Heavy metals are defined as metals with a specific gravity above 5.0 and atomic weight between 63.5 and 200.6. Some of them are present in the human body and are fundamental for several biological processes, but when their concentration exceeds upper limit values, they become dangerous for human health. Their harmful effects on humans depend on the dosage, rate of emission and period of exposure [2,4].

The level of toxicity for the human body of some heavy metals follows the order Co < Al < Cr < Pb < Ni < Zn < Cu < Cd < Hg [5].

Table 1 reports the main harmful effect of some heavy metals, used in industrial activities, and their upper limit concentration in drinking water according to the the World Health Organization (WHO) [4].

Water treatments for the removal of dyes and heavy metals include biological processes (in aerobic or anaerobic conditions), chemical processes (such as ozonation, coagulation and precipitation, ion exchange, electro-coagulation) and physical processes (flotation, reverse osmosis, adsorption) [2]. Among these processes, adsorption is considered one of the most effective and competitive and is widely used in industrial applications. In an adsorption process, the substances of a fluid, liquid or gas bind to the external and interior surfaces of the adsorbent material. The main advantages are low cost, high efficiency, ease of operation and implementation, the possibility to use several solids as adsorbent materials, and the possibility to recover the adsorbent and the adsorbate [6]. It is interesting to compare the costs of the main technologies for wastewater treatment (reverse osmosis, ion exchange, electro-dialysis, electrolysis) with those of the adsorption process: the first range from 10 to 450 USD/m^3^ treated water, while adsorption ranges from 5.0 to 200 USD/m^3^ [7]. Depending on the nature of the adsorbent and on its textural properties, the adsorbent–adsorbate interaction can be physical, also called van der Waal’s adsorption (adsorption into the adsorbent pores), and/or chemical (ion exchange or acid–base reactions). Generally speaking, besides low cost and availability, an adsorbent material should possess chemical and mechanical stability and good textural properties (high surface area and pore volume, suitable functional groups) in order to guarantee high adsorption efficiency, fast kinetics, recovery and reusage [2,7,8].

As far as the operation mode is concerned, adsorption processes are generally carried out by batch experiments at the lab scale, while a fixed-bed operation mode is suitable for large scale treatment applications, allowing the treatment of large water volumes in small physical areas, without requiring additional separation operations [2,9]. Moreover, fixed-bed columns for industrial processes have higher residence times and better heat and mass transfer characteristics than batch reactors [9].

Adsorption isotherm modeling is used to describe the interaction between adsorbate and adsorbent and how the adsorbate is distributed between the solution and the solid phase at the equilibrium state. Experimental data can be fitted by several adsorption isotherm models, among them Langmuir, Freundlich, and Sips [10,11,12,13]. The Langmuir model is based on the formation of an adsorbate monolayer at the outer surface of the adsorbent. According to this model, all the adsorption sites are energetically equivalent and identical.

Differently, the Freundlich isotherm is based on a heterogeneous adsorption with different adsorption energies.

The Sips model consists of the combination of the Langmuir and Freundlich isotherm models. The Sips model reduces to the Freundlich isotherm at low metal ion concentrations, while at high metal concentration it follows the Langmuir isotherm.

Kinetic studies also play an important role in the characterization of an adsorption process, since they provide information about the uptake rate of the adsorbate and, hence, on the efficiency of the adsorption process. Experimental data can be validated, among others, by pseudo-first order, pseudo-second order or intraparticle kinetic models [14,15].

According to Crini et al., adsorbent materials can be classified as conventional and unconventional materials [8,9,16].

Among conventional adsorbents, activated carbons, inorganic materials such as activated aluminas, silica gel, zeolites, and ion-exchange resins are included [17,18]. Unconventional adsorbents include materials from agricultural and industrial waste, natural materials such clays, biosorbents such as chitosan, and miscellaneous adsorbents such as alginates [8,9,19,20,21,22,23,24]. These sorbents have the advantage of being low-cost or free of cost materials. In addition to these, other unconventional materials have been investigated, among them graphene, carbon nanotubes, Metal Organic Frameworks, and layered zirconium phosphates [25,26,27,28,29,30].

Layered zirconium(IV) phosphates are a well known class of inorganic materials, whose intercalation and ion exchange properties are widely investigated [31]. Their chemical and thermal stability, the availability of several synthetic procedures, the possibility to easily tune its properties by changing the synthetic approach, the possibility to easily bind functional groups on the layer surface and their ability to uptake cations, both inorganic and organic, and basic molecules stimulated the interest in this class of compounds as potential and unconventional materials for the removal of heavy metals and dye from water. As proof of this, several papers reported their use for the removal of heavy metals and dyes from water. Nevertheless, to the best of our knowledge, specific reviews dedicated to the use of zirconium phosphates in pollution remediation were not found in the literature, except for the paper by Pandith et al., published in 2020 [32], that, however, was dedicated, among other things, just to the metal uptake by zirconium phosphate, while concerns about dye uptake were not addressed.

On the basis of these considerations, it seemed of interest for the scientific community to gather/collect the recent studies on water treatment by using zirconium phosphates for pollutants’ removal, specifically heavy metal cations and dyes.

## 2. Structure of Zirconium Phosphate

The history of zirconium phosphate compounds started in the 1950s, when the first papers on their use as sorbents for metal cations were published [33,34,35].

Zirconium phosphates can be classified according to their crystal structure. One-dimensional, two-dimensional and three-dimensional structures are known [36] and, among these, α-type layered structures are the most studied as cation exchangers and intercalation hosts [31,37,38,39].

Zirconium phosphate of α-type (Zr(HPO_4_)_2_ H_2_O, ZrP) is made of layers in which the Zr atoms bond monohydrogenphosphate groups, with the P-OH groups pointing in the interlayer region, alternatively below and above the main plane. Six phosphates are coordinated to a Zr atom through oxygen atoms, and the oxygens of each phosphate are shared with three Zr atoms. Water molecules are located between the layers in six-sided cavities and form hydrogen bonds with the P–OH groups of the same layer [31].

The interlayer distance of ZrP is 7.56 Å and the presence of protogenic–OH groups is responsible for its cation exchange properties, and both inorganic and organic cations can be inserted in the interlayer region [31]. 

Microcrystalline ZrP has an ion exchange capacity of 6.6 meq/g. Generally, about half of the protons are exchanged at relatively low pH, with the formation of a half-exchanged phase which is converted in the fully exchanged phase at higher pH (neutral or slightly basic). Moreover, the protons of the P–OH groups can react with bases, leading to the intercalation of basic molecules [31].

ZrP is a highly versatile material, whose properties are strictly connected to its structure and degree of crystallinity which can be controlled by changing the synthetic conditions.

Amorphous, microcrystalline and nanocrystalline materials were obtained in different conditions. Amorphous gel materials were generally obtained by reaction between phosphoric acid and zirconyl chloride in water, without using any complexing agent for Zr(IV) [33,34,35,40], while post treatments of amorphous ZrP produced powders, granules and thin films [41,42]. Sol–gel and template methods were also developed in order to produce ZrP materials with different morphologies and porosity [43,44,45,46,47,48,49,50].

By refluxing amorphous ZrP in the presence of concentrated phosphoric acid, microcrystalline materials were obtained [51]. Alternatively, microcrystalline ZrP was prepared by Alberti et al. through hydrothermal synthesis by the slow decomposition of ZrF_6_^2-^ complexes in the presence of phosphoric acid [52]. Chuah et al. proposed a modification of this hydrothermal method in the presence of small amounts of fluoride ions, leading to platelets and rods [53]. Alternatively to fluoride, oxalate anions were used as complexing anions for Zr(IV) [54].

Nanocrystalline ZrP was prepared by Pica et al. by using a quick procedure, consisting of mixing, at room temperature, zirconyl propionate and concentrated phosphoric acid in aliphatic alcohols [55]. A gel product was obtained, containing ZrP nanoparticles with an average planar size of tens of nanometers. By heating the gels to dryness, nanocrystalline ZrP, consisting of nanoplatelets with an average planar size of hundreds of nanometers, was obtained for phosphate to zirconium molar ratios higher than 4.

Amorphous, microcrystalline and nanocrystalline ZrP were investigated as sorbent materials for heavy metals and dyes, and in the following sections these applications are discussed.

## 3. Removal of Heavy Metals

Amorphous ZrP, prepared by the reaction of zirconyl chloride, dissolved in HCl solution, with phosphoric acid at room temperature, was employed by Pan et al. for the removal from water of heavy metal cations, specifically lead, zinc and cadmium [40]. The ion exchange properties of ZrP were evaluated, finding that about half of the total amount of protons, corresponding to 3.04 meq of H^+^ per gram, is released into the solution at pH < 7, which is indispensable for the removal of heavy metals because most of heavy metals precipitate in solution at alkaline condition. Batch sorption tests, carried out at 30 °C by adding ZrP to a solution containing the selected heavy metals, proved that protons are stoichiometrically exchanged with the heavy metal cations, according to the following reaction:

(1) ZrHPO42+12M2+⇆ ZrM12HPO42+ H+

Moreover, it was found that the sorption capacities of the three heavy metal ions decreased with increasing the pH solution, in agreement with the Le Chatelier Principle. The affinity order of ZrP for the cations, determined by sorption isotherms, was: Pb^2+^ > Zn^2+^ ≈ Cd^2+^. The authors speculated that, among other factors, this order could also be affected by the hard and soft properties of the involved species, according to the hard and soft acids and bases (HASB) theory [56,57,58]. Lead ion, as a Lewis acid, lies between hard and soft ones and preferably interacts with orthophosphate ion, a Lewis base also in borderline, while cadmium, a soft acid, presents weaker interaction with ZrP and consequently exhibited low selectivity.

Regeneration efficiencies, evaluated by the treatment of the cation-exchanged ZrP samples with HCl, were 95.3%, 99.6%, and 99.9% for ZrP loaded with Pb, Zn, and Cd, respectively. However, it should be pointed out that the synthetic procedure used for the synthesis of amorphous ZrP produced ultrafine particles that cannot be directly used at large scale in column operation, due to the unacceptably high pressure drop.

To overcome this problem, hybrid sorbents, made of ZrP particles immobilized onto porous materials or dispersed in a polymer matrix, were proposed for large-scale applications.

In the following, ZrP combined with polymers or other inorganic materials will be described.

### 3.1. ZrP–Polymer Composites

Several examples of sorbents based on polymer composites, consisting of ZrP particles dispersed in a polymer matrix, are reported in the literature. Charged and uncharged polymers were used, also offering, among other things, the advantage to in situ precipitate ZrP particles.

Pan et al. prepared a hybrid sorbent by loading ZrP particles onto a polystyrenesulfone cation exchanger D-001 [59]. D-001 was selected as the host material due to the presence of a negatively charged sulfonic acid group, which would improve the permeation of the targeted metal ions. As a matter of fact, D-001 is recommended by the US Environmental Protection Agency as the best available material for heavy metal contamination control [59].

The composite material (hereafter ZrP-001) was prepared by adding D-001 to a solution of ZrOCl_2_ in HCl. After the evaporation of HCl, H_3_PO_4_ was added to precipitate ZrP. The ZrP wt% was 33% and its incorporation onto D-001 caused a decrease in the surface area and pore volume of the polymer matrix.

The ZrP-001 composite presented two different exchangeable sites for the uptake of heavy metals: the sulfonic group of D-001 and the phosphate groups of ZrP, according to Equations (1) and (2):

(2) $$R-{\mathrm{SO}}_{3}H+1/2M^{2+}⇆{\;\mathrm{RSO}}_{3}M_{0.5}+{\;H}^{+}$$

The ion-exchange capacity value of the composite, determined experimentally, was 3.20 meq/g, which was less than that calculated (4.07 meq/g) at neutral pH, but slightly higher that that found for ZrP in ref. [40].

Both batch and column sorption tests were carried out. From batch experiments, the uptake of the heavy metals at pH less than 0.5 was negligible, in agreement with the fact that the used ZrP-001 might be regenerated by strongly acidic solution, while at higher pH solution (under acidic or neutral conditions) the uptake was more favourable and higher than that of D-001; the same preference order found in ref. [40] was observed (Pb^2+^ > > Zn^2+^ ≈ Cd^2+^).

Fixed-bed column adsorption tests for the heavy metals were carried out in the presence of competing cations (Na^+^, Ca^2+^, and Mg^2+^) and confirmed the more efficient sorption of heavy metals on ZrP-001 than on D-001, despite its lower ion exchange capacity (3.2 vs. 4.2 meq/g), and the higher selectivity of ZrP-001 for heavy metals with respect to innocuous cations. Moreover, less than 1% loss of ZrP in ZrP-001 beads was detected after five-column cycles.

A mesoporous polystyrene (MPS) matrix was also used to immobilize ZrP [60]. According to this procedure, the MPS host was first prepared by a flash freezing method, which allowed the acquisition of spherical beads of 1.5–2.0 mm in diameter, with abundant uniform pores of around 7.9 nm. MPS was then immersed into ethanol solutions containing ZrOCl_2_, followed by evaporation to promote the diffusion of [Zr(OH)_2_(H_2_O)_4_]^2+^ cations inside the nanopores of the MPS host. The beads were then incubated with H_3_PO_4_ solution to induce the in situ growth of the ZrP nanoparticles. The ZrP loading in ZrP@MPS was about 7% in Zr mass and ZrP nanoparticles with an average diameter of 6.5 nm were well dispersed in ZrP@MPS. XRD and ^31^P-MAS NMR analyses revealed the coexistence of α-ZrP and γ-ZrP and this was a quite surprising result, since γ-ZrP is normally obtained by boiling, under reflux conditions, an amorphous gel for several weeks [61].

The authors first evaluated the adsorption reactivity of both α-ZrP and ZrP@MPS toward Pb^2+^. They found that pure MPS exhibited negligible adsorption toward heavy metals; moreover, the adsorption distribution coefficients *K*_d_ of ZrP@MPS were much higher (10–90 times higher) than those of α-ZrP. On the basis of the XPS studies, the authors proposed that ZrP@MPS adsorbed Pb^II^ mainly through the inner-sphere coordination rather than through an electrostatic interaction. They also examined the selective adsorption of the sample toward a series of heavy metal cations (Pd, Cd, Ni) in the presence of much higher concentrations of competing mineral cations, e.g., Ca^2+^, in real contaminated water. With respect to other systems [59], the adsorption capacities toward heavy metal cations are less disturbed by Ca^2+^ (≈100%, >70%, and >40% for Pb^2+^, Cd^2+^, and Ni^2+^, respectively), even at high Ca concentrations; the authors speculated that the higher affinity of the sorbent material toward heavy metals was mainly due to the kind of interaction between them: the specific inner-sphere coordination interaction between ZrP@MPS and heavy metal cations favoured the selectivity toward heavy metals more than nonspecific electrostatic attractions. ZrP@MPS was also used to treat simulated polluted water containing Pb^2+^ in a continuous column mode. A commercial D001 sample was used in parallel. The results showed that the D001 column produced clean water with a mass of ≈1500 times over the adsorbent mass, while the ZrP@MPS column was able to generate clean water with a mass of 7200 times over the adsorbent mass in each run. Finally, the ZrP@MPS absorbent material could be easily and effectively regenerated by treating the fully adsorbed sample with hydrochloric acid.

Polysulfide (PSF) capsules containing zirconium phosphate were prepared by Li et al. for the removal of Pb^2+^ ions from aqueous solutions [62]. Amorphous ZrP was prepared according to ref. [40], while PSF@ZrP capsules were prepared by the phase inversion precipitation technique. N-methylpyrrolidone was used as a solvent to dissolve PSF and disperse ZrP, while sodium dodecylsulfate in ethanol–water solution was used to form the PSF@ZrP capsules. Capsules with different PSF/ZrP mass ratios were prepared. The characterization of the PSF@ZrP capsules revealed the amorphous nature of ZrP and that their surface area was lower than that of the pure PSF capsules, due to the blockage of a fraction of the pores by the inorganic material. SEM images showed the presence of spherical capsules with a rough surface and with ZrP particles uniformly dispersed from the outer surface to the inner part of the capsules.

Adsorption experiments were carried out by using a batch method, and the effect of pH, contact time, initial concentration, temperature and competing ions was studied. Pure PSF capsules had an extremely low affinity to Pb^2+^. The lead adsorption significantly increased from 12 to 62 mg g^-1^ with increasing the ZrP mass ratio in the capsules from 1:0 to 1:1 (PSF to ZrP). However, a further addition of ZrP up to 1:1.5 (PSF to ZrP) provoked a decrease in the adsorption capacity for Pb^2+^. On the basis of these results, the authors selected the capsule with PSF/ZrP mass ratio = 1 to continue the study. The optimal pH condition for Pb^2+^ uptake was the original pH of the solution, that is, 5.75. Stronger acidic conditions did not favor the adsorption, suggesting that the PSF@ZrP capsules may be regenerated in strong acidic conditions. The equilibrium time was independent of the initial Pb^2+^ concentration. Moreover, the initial rate of adsorption increased with increasing the initial Pb^2+^ concentration because of the increased driving force, resulting from the difference between the Pb^2+^concentration in the solution and onto the capsules. Although the amount of Pb^2+^ adsorbed at equilibrium increased from 56 to 102 mg g^−1^ with increasing the Pb^2+^ concentration from 100 to 300 mg L^−1^, the removal percentage of Pb^2+^ from solution decreased from 56% to 34%.

PSF@ZrP capsules showed satisfactory affinity to bind Pb^2+^ with respect to other similar reported sorbents, such as alumina and modified alumina [63,64]. Furthermore, the maximum uptake amount of the PSF@ZrP capsules is over hundred times larger than that of ZrP modified silica [65].

The effects of competing cations such as Na^+^ and K^+^ on the Pb^2+^ uptake onto the PSF@ZrP capsules were also studied, finding that the amount of Pb^2+^ sorbed onto the PSF@ZrP capsules was slightly influenced when the concentration of the coexisting ions was low, while it decreased as the concentration of Na^+^ and K^+^ was hundreds of times more than that of Pb^2+^. Finally, adsorption re-generation cycles were performed six times with no significant loss of adsorption capacity.

Macroporous polystyrene resins, with different functional groups, i.e., -CH_2_Cl, -SO_3_H, and -CH_2_N^+^(CH_3_)_3_, were also used to encapsulate ZrP nanoparticles, thus obtaining three nanocomposite adsorbents (denoted as ZrP–Cl, ZrP–S, and ZrP–N, respectively) for lead removal from water [66].

The host polymers with -SO_3_H and -CH_2_Cl functional groups were, respectively, prepared from polystyrene–divinylbenzene copolymer (St–DVB) by reaction with concentrated sulforic acid (hereafter S–St–DVB) and chloromethyl ether with zinc chloride as the catalyst (hereafter Cl–St–DVB), while -CH_2_N^+^(CH_3_)_3_ groups were introduced by the reaction of Cl–St–DVB with trimethylamine solution (hereafter N–St–DVB).

Polymer composites were prepared by immersing Cl–St–DVB, S–St–DVB, or N–St–DVB beads in ethanol solution containing different amounts of ZrOCl_2_. Then, each ZrOCl_2_-loaded polymer was treated with H_3_PO_4_ solution. The ZrP wt% in each composite was about 20 wt%.

TEM images of the three nanocomposites showed that both ZrP–S and ZrP–N consisted of ZrP nanoparticles with an average size of about 10–40 nm. Obvious particle agglomerates were observed within ZrP–Cl (20–140 nm). The authors suggested that the presence of negatively or positively charged functional groups in the polymeric supports was more favourable than the neutral chloromethyl group to form small ZrP nanoparticles. In other words, nanoparticle dispersion or aggregation is greatly associated with both van der Waals attraction and electrostatic double-layer repulsion interaction between the particles. The repulsion interactions between adjacent nanoparticles generally dominate the extent of their aggregation and particle size distribution. As proof of that, ZrP–N and ZrP–S had similar absolute values of zeta potential, which were higher than that of ZrP–Cl, in agreement with the improved ZrP dispersion of the two former nanocomposites. Sorption isotherms of Pb(II) by the three composites, in the presence of Ca(II) as a competing cation, showed that the sorption capacities were in the sequence of ZrP–S > ZrP–N > ZrP–Cl. The observed trend can be explained by considering both the ZrP particle size and the nature of electrical charge immobilized on the polymer surface.

The authors also evaluated the mechanical properties of the composites in light of their importance to improve their feasibility for practical application. On the basis of the sorption tests, S–St–DVB was selected as the host material to fabricate several composite adsorbents with different amounts of ZrP. Then, the mechanical strength of the resulting composites was examined in terms of compressive strength and compared with the pure S–St–DVB host polymer, finding that the maximum compressive strengths (MCS) of all the resulting nanocomposites were greatly improved with respect to that of the host polymer; the optimum nano-ZrP loading was about 5 wt%.

Alam et al. validated the ion exchange process on a composite cation exchanger based on nylon 6,6 and ZrP for a practical application in the wastewater treatment process [67]. ZrP was prepared by the reaction of zirconyl oxychloride with H_3_PO_4_ at pH = 1. Nylon 6,6 gel, obtained by treatment with concentrated formic acid, was added to ZrP and mixed thoroughly with constant stirring. Composite cation exchanger particles of mean radii of 125 nm in H^+^ form were used to evaluate various kinetic parameters with heavy toxic metal pollutants such as lead, cadmium, zinc and copper. Kinetic studies were carried out at various temperatures under particle diffusion controlled phenomena. Various kinetic parameters such as self-diffusion coefficient (Do), energy of activation (E_a_), and entropy of activation (ΔS*) were evaluated to validate the ion exchange process. It was found that equilibrium is attained faster at a higher temperature with the particle diffusion controlled phenomenon. Moreover, the diffusion coefficient for the four heavy metal ions studied on ZrP composites followed the order Cu^2+^ > Pb^2+^ > Zn^2+^ > Cd^2+^, also confirmed by the highest ΔS* value for the H(I)–Cu(II) exchange.

Polyaniline (PANI) was used to prepare composite films loaded with ZrP platelets for potential-triggered adsorption of Pb^2+^ ions [68]. A highly crystalline α-ZrP was first synthesized by the hydrothermal method and then exfoliated by tetrabutylammonium hydroxide. ZrP nanosheets/PANI was deposited on a carbon nanotube modified Au electrode in aqueous solution by the electropolymerization of aniline, in the presence of ZrP, by the cyclic voltammetry (CV) method. The presence of CNTs had the advantage of increasing the roughness of the electrode surface, thus promoting the electrodeposition of ZrP/PANI. At the reduction state of CV, a quinonoid amine (=N-) of PANI seized the proton from the α-ZrP nanosheet to form benzenoid amine (NH) [69], leading to PO^-^ Pb^2+^ interactions. The incorporation and release of ions in the hybrid film was studied by the electrochemical quartz crystal microbalance (EQCM) technique, by in situ detection of the mass change of the film because of the charging and discharging associated with the ion exchange process between the film and electrolyte. It was found that, while the pure PANI film behaved as an anion exchanger, the α-ZrP/PANI hybrid film behaved as a cation exchanger. The mass increased when the composite film was reduced due to the insertion of Pb^2+^ ions into the film to neutralize the reduction centers. During the oxidation of the film, the Pb^2+^ ions were released into the solution to maintain charge balance and resulted in the mass decrease. A set of CV/EQCM experiments was conducted to quantify the preferential Pb^2+^ selectivity in the presence of Ni^2+^, Cd^2+^, Zn^2+^ and Co^2+^ ions. The ion adsorption selectivity follows the sequence of Pb^2+^ > Ni^2+^ > Co^2+^ > Cd^2+^ > Zn^2+^, and the adsorption capacity towards Pb^2+^ ions was at least four times higher than that of other heavy metal ions.

Chen and coworkers fabricated a zirconium phosphate modified polyvinyl alcohol-polyvinylidene fluoride, (PVA)-PVDF, membrane for lead removal [70]. The zirconium ions and PVA were firstly coated onto a PVDF membrane through crosslinking reactions with glutaraldehyde, which was then modified by phosphate. Lead adsorption was studied by batch experiments. It was found that lead adsorption increased with an increase in pH up to pH = 5.5. The adsorption isotherms, studied at pH 5.5, showed that the experimental data were better described by the Langmuir equation than the Freundlich equation, suggesting that the adsorption sites onto the membrane are relatively homogenous. The maximum adsorption capacity was 121 mg-Pb/g at pH 5.5 for lead simulated water (prepared by DI water). A good selectivity in the adsorption towards lead with respect to zinc was also found, with a selectivity coefficient of lead/zinc around 10. Four cycles of adsorption and desorption were conducted to test the reusability of the modified PVDF membrane. After four cycles, the adsorption capacity was still as high as 95.6% of the virgin membrane in the first cycle.

An interesting paper by Hasan et al. reported the use of cellulose membranes coated with α-ZrP nanoparticles (α-ZrP-n) for the removal of heavy metals from wastewater [71]. The composites were prepared by spraying an aqueous dispersion of commercial ZrP nanoparticles, with an average particle size diameter of 100 nm, onto the surface of the pure cellulose membranes. Leaching tests proved that ZrP strongly interacted with cellulose, since no traces of Zr were found after the immersion of the membrane in water for 24 h. Moreover, mechanical tests showed that the presence of ZrP nanoparticles did not negatively affect the mechanical properties of cellulose fibers, while a decrease in the membrane porosity was observed with increasing the α-ZrP-n concentration, also leading to a reduction in water flux.

The synthetic wastewater sample containing heavy metals (Ni, Zn, Pb, Cu) was filtered using both the pristine cellulose and α-ZrP-n coated membranes using vacuum filtration. At pH = 7, all composite membranes exhibited a removal percentage of all heavy metals higher than that of pure cellulose. The best results were obtained with Pb^2+^, reaching a percentage of removal of about 60% with the membrane containing 1 wt% of α-ZrP-n.

### 3.2. Other ZrP Based Materials

In order to improve the textural properties ZrP, making it more suitable for large scale applications, several synthetic approaches were developed. In some cases, ZrP was combined with other inorganic materials and synergistic effects were observed.

Parida et al. proposed a titania pillared zirconium phosphate to remove hexavalent chromium from aqueous solutions by solar radiation [72]. Titania is a well known photocatalyst under UV light acting as a reducing agent able to reduce the harmful Cr(VI) to the less harmful Cr(III). Titania pillared ZrP was prepared from Na-exchanged ZrP following the procedure reported by Yamanaka et al. [73]. First, titania sol was prepared by the hydrolysis of titanium(IV) isopropoxide by HCl. Then, an aqueous suspension of sodium-exchanged ZrP was added to the sol. The suspension was filtered, washed and calcined at 500 °C. Composites with titania loadings in the range 1–10 wt% were prepared.

The photo-reduction of Cr(VI) was performed in batch. The solution was exposed to sunlight in closed flasks at room temperature with constant stirring. The effect of EDTA and 4-nitrophenol, as sacrificial electron donors, was also studied, keeping all other parameters fixed.

It was found that the photo-reduction of hexavalent chromium was strongly dependent on pH. The highest reaction rate was obtained at acidic pH (1–2). Moreover, by increasing the titania loading up to 2 wt%, the initial rate of the Cr(VI) photoreduction increased, and this could be due to the fact that an increase in titania resulted in higher surface area, which allowed more Cr(VI) species to be adsorbed on the surface, thus facilitating the photo-reduction process. Differently, the rate of photo-reduction of Cr(VI) decreased with increasing the initial Cr(VI) concentration. The percentage of photoreduction reached 100% at a lower Cr(VI) concentration (<10 mg/L). By increasing the catalyst dose or the irradiation time, the rate of photo-reduction of Cr(VI) initially increased; thereafter, it remained almost constant.

By comparing the effects of sacrificial electron donors such as EDTA and 4-nitrophenol, a significant effect was observed for EDTA. Moreover, experiments were also carried out by varying the atmosphere of the photo-reduction process by bubbling N_2_, O_2_ and air, finding that, among them, N_2_ had the most significant effect on the photo-reduction, while the dissolved oxygen had no effect or very negligible effect on the photo-reduction process.

**Figure 1:**
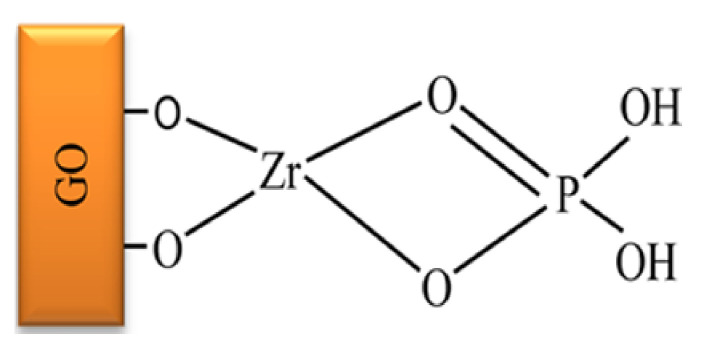
Schematic representation of ZrP bonded to the surface of graphene oxide (GO) ([74], with permission of ACS).

Graphene oxide–zirconium phosphate (GO–ZrP) nanocomposite was proposed by Pourbeyram as an adsorbent for the removal of Pb(II), Cd(II), Cu(II), and Zn(II) from aqueous solutions [74]. The GO–ZrP composite was prepared by the dropwise addition of zirconium chloride to a GO suspension under sonication, followed by the addition of sodium dihydrogen phosphate. The adsorption of phosphate on the surface of GO–Zr was represented schematically as follows (Figure 1):

The transmission electron microscopy (TEM) images of the GO–ZrP nanocomposite revealed the presence of ZrP nanoparticles of ∼1–2 nm in diameter. The nanoparticles were well distributed, with a high density on the GO surface and interparticle distances in the range of ∼3–5 nm.

The adsorption of heavy metals was performed in a batch experiment. GO–ZrP nanocomposite was added to a solution containing Pb(II), Cd(II), Cu(II) and Zn(II), at desired initial concentrations. The effect of pH on the adsorption of heavy metals was studied in the pH range of 1–8. At the range of pH 3–6, high and relatively constant adsorption capacity was observed. It was found that, for all metals, the adsorption occurred in two different steps. During the first 10 min, the adsorption increased rapidly. After that, adsorption increased gradually and finally reached equilibrium after 20 min. After adsorption, a tendency of the nanocomposite to agglomerate and precipitate was observed. Moreover, the maximum adsorption capacity of GO was much lower than that for the GO–ZrP nanocomposite. On the other hand, on the GO–Zr nanocomposite, no adsorption of heavy metals was observed under the same conditions.

The amount of heavy metals adsorbed on the nanocomposite increased by increasing the initial concentration of the heavy metals in the range of 10–200 ppm, and then, the sorbent was finally saturated by a relatively constant amount of the heavy metals (∼200 ppm). The results of batch experiments indicated that the maximum adsorptions for Pb(II), Cd(II), Cu(II), and Zn(II) at pH 6 were 363, 232, 329, and 252 mg g^−1^, respectively, corresponding to 3.5, 4.1, 10.4, and 7.7 meq g^−1^, respectively. Moreover, the removal of heavy metals at lower amounts of sorbent took more time than that at higher amounts.

**Figure 2:**
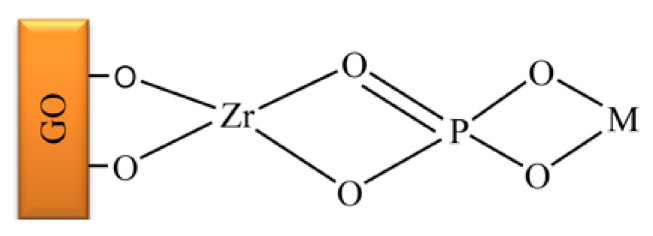
Schematic representation of the adsorption of a metal ion onto GO–ZrP ([74], with permission of ACS).

Adsorption isotherms showed that the process occurred at the functional groups/binding sites on the surface of the GO–ZrP nanocomposite, according to a monolayer adsorption. It was found that the adsorption capacity of the GO–ZrP nanocomposite was significantly higher than that of the most part of adsorbents. A possible configuration of the adsorption of metal ions (M) can be represented schematically as follows (Figure 2):

The desorption study showed that the GO–ZrP nanocomposite was effectively regenerated (∼100%) by the treatment of metal ion-loaded nanocomposite with 3 M HCl for ∼10 min. The nanocomposite was easily separated from the system via centrifugation. The high adsorption efficiency performance was maintained after being used for at least five cycles.

Besides inorganic compounds, organic molecules with high metal affinity were combined with ZrP to fabricate composite adsorbents with improved properties. On this regard, the ability of crown ethers to recognize cations in a selective fashion has been known since the 1970s [75], as well as their ability to interact with zirconium phosphates through covalent and uncovalent interactions [76,77]. Peng and coworkers fabricated a new organic–inorganic layer α-ZrP composite by the intercalation of 4-amino-benzo-18-crown-6 (AM–ZrP) to remove radioactive ^90^Sr from solution [78]. This crown ether was chosen since it has a strong complexing ability for Sr^2+^. α-ZrP was prepared by the fluorine reflux method. The intercalation of 4-amino-benzo-18-crown-6 was carried out on ZrP pre-intercalated with butylamine (BU–ZrP) in order to enlarge the interlayer region and promote the intercalation of crown ether. The authors speculated that the arrangement of 4-amino-benzo-18-crown-6 in the layer structure of α-ZrP comprised double inclined layers, which is one of the best modes that could load the maximum amount of 4-amino-benzo-18-crown-6.

The AM–ZrP composite exhibited excellent stability under acid and radioactive conditions: this is an important key factor for application in nuclear wastewater disposal, as most ^90^Sr exists in acidic fission solution produced by dissolving uranium targets in strong HNO_3_ solution. The intercalation of crown ether in ZrP also efficiently decreased its loss in the adsorption process. 

The adsorption of Sr^2+^ by α-ZrP, BU–ZrP and AM–ZrP was measured in aqueous solution. Isotherm studies proved that the adsorption data were best fitted by the Langmuir model, indicating that Sr^2+^ adsorbed onto α-ZrP, BU–ZrP and AM–ZrP materials forms a monolayer. The maximum adsorption capacity of Sr^2+^ onto α-ZrP, BU–ZrP and AM–ZrP is 63.14 mg g^−1^, 162.36 mg g^−1^ and 320.14 mg g^−1^, respectively, at pH = 5. Moreover, adsorption kinetics of Sr^2+^ on α-ZrP, BU–ZrP and AM–ZrP showed that about 90% of Sr^2+^ ions were removed within the first 60 min, with equilibrium gradually reached in approximately 150 min. It is noteworthy that the adsorption capacity of AM–ZrP is higher than that of other similar zirconium adsorbents [78].

The study of Sr^2+^ uptake by AM–ZrP in the presence of other metal cations from Li, Na, K, Mg, Cs, Pd, Mo, Zr, Ca, and Ba proved its excellent selectivity for Sr^2+^, which is mainly due to the complexing action, rather than ion exchange and surface physical adsorption. Unfortunately, the authors did not report information about the performances of AM–ZrP in column separation and nuclear wastewater disposal processes.

Melamine is a N-rich chelating molecule, which was investigated in heavy metal capture [79,80]. El-Shall et al. studied melamine zirconium phosphate (M–ZrP) adsorbent for the extraction of heavy metals from polluted water [81]. M–ZrP was prepared in two steps: first, melamine phosphate (MP) was prepared by the acid–base reaction between phosphoric acid as a proton donor and melamine as a proton acceptor. Then, zirconium tetrachloride was added to form amorphous ZrP. TEM images show that M–ZrP consisted of small particles connected together in mesoporous structures with a Brunauer–Emmet–Teller (BET) surface area of 320 m^2^ g^−1^.

The adsorption capacity of M–ZrP was tested for Pb(II), Hg(II) and Cd(II). It was found that the M–ZrP adsorbent shows exceptionally high adsorption affinity for Pb(II) with a capacity of 681 mg g^−1^ and 1000 mg g^−1^ using an adsorbent dose of 1 g L^−1^ and 2 g L^−1^, respectively. The high adsorption capacity is also coupled with fast kinetics with an equilibrium time, required for the 100% removal of Pb(II), of the order of seconds and minutes, depending on the metal concentration.

In a mixture of six heavy metal ions, the removal efficiency was 100% for Pb(II), 99% for Hg(II), Cd(II) and Zn(II), 94% for Cu(II), and 90% for Ni(II) at a lower concentration, while at a higher concentration the removal efficiency for Pb(II) was 95% compared to 23% for Hg(II) and less than 10% for the other ions.

Despite the difficulties of using pure ZrP as a metal sorbent in a continuous operation mode on a large scale, it is noteworthy that researchers worked to develop strategies to optimize the particle morphology and textural properties of ZrP in order to improve, on one hand, its uptake capacity and, on the other hand, to make it more suitable for large scale applications.

Nakanishi et al. fabricated hierarchically porous ZrP monoliths combining micrometer order macropores and nanometer order mesopores [82]. Monoliths were prepared by a sol–gel process in which zirconium oxychloride reacted with phosphoric acid in the presence of suitable amounts of poly(ethylene glycol) (PEO) and polyacrylamide (PAAm) in order to induce phase separation during the sol–gel process. A macroporous co-continuous structure was obtained only when both polymers were added together into the starting solution. Mesopores, with an average mesopore size of 5 nm, were found in the supercritically dried ZrP monoliths. A syringe device with the tight-fit ZrP monolith was designed to efficiently treat contaminated water under a continuous flow condition. In each run, a metal salt solution was introduced through the syringe device to investigate the efficiency of ion adsorption, even at high ion concentrations. Altogether, eight kinds of metals were used, including Ag^+^, Cs^+^, Sr^2+^, Cu^2+^, Zn^2+^, Pb^2+^, Cd^2+^ and Fe^3+^. The ZrP monolith showed the highest selectivity for Cu^2+^, Pb^2+^ and Fe^3+^ and the lowest for Ag^+^.

An egyptian zircon mineral was used by Ali for the synthesis of ZrP and its use for uptaking uranium was studied [83]. Zirconium chloride solution was obtained by Rosetta zircon and used for the synthesis of ZrP by reaction with sodium dihydrogen phosphate. The desired particle size (30–60 mesh) was selected by grinding and sieving. An amorphous ZrP material, with P:Zr molar ratio = 2:1, was obtained and used for U(VI) adsorption tests. It was found that U(VI) adsorption increased with increasing pH and reached a maximum (98.5%) at pH 5, and then declined sharply as pH was further increased. Moreover, 30 min was chosen as the optimum time where adsorption equilibrium reached about 98%. Kinetic studies showed that the adsorption process of U(VI) on ZrP can be expressed by a pseudo-second-order kinetic model. Thermodynamic studies revealed that the optimum temperature for U(VI) uptake was 298 K, and the positive value of ΔS reflected the good affinity of uranium ions towards the sorbent and the increasing randomness at the solid–solution interface during the adsorption process. U(VI) uptake was also studied in the presence of other metal ions (Cu, Ni, Fe, Th, and Pb). Moreover, it was found that the uranium uptake efficiency slightly decreased in the presence of competing ions, but it remained preferred with respect to the other cations. Adsorption isotherm studies revealed that the Langmuir isotherm described the system more adequately than the Freundlich isotherm and, according to that model, the adsorption occurred uniformly on the active sites of the sorbent, and once a sorbate occupied a site, no further sorption could take place at that site. A case study was carried out by using a Gattar leach liquor solution contacted with ZrP for 30 min at room temperature and the pH adjusted at 2–3. After pH adjustment and equilibration, the recovered solution was analyzed for the uranium concentration and it was found that the adsorption efficiency of ZrP was about 70%. It is also noteworthy that ZrP prepared from zircon mineral was more efficient than ZrP prepared from H_3_PO_4_ and ZrOCl_2_^.^8H_2_O (98% vs. 76.5%).

Pandith et al. prepared agglomerated spherical alpha zirconium phosphate nanoparticles by a facile and rapid microwave hydrothermal approach in the absence of any complexing or structure directing agent and tested their efficiency in the removal of radioactive ^137^Cs^+^ and ^90^Sr^2+^ ions from aqueous systems [84]. FTIR, X-Ray diffraction and BET analysis confirmed that the formation of α-ZrP occurred, with an average crystallite size of 3–4 nm, a mesoporous structure and a specific surface area of about 500 m^2^ g^−1^. The removal of radioactive ions was tested by non-competitive batch measurements in acidic aqueous media (pH in the range 3.0–6.0, generally found in nuclear wastewater). It was found that about 98.3% of ^90^Sr^2+^ was removed from the aqueous solution within 160 min of contact time. Although the adsorption of Cs^+^ reached equilibrium within 40 min, the removal % was only 76.5%. The removal of metal cations was observed to depend on the hydrated radii and on the charge on the exchanging ions.

Chuah et al. prepared a two-dimensional disodium zirconium phosphate, Zr(NaPO_4_)_2_·H_2_O (hereafter indicated as α-Na_2_ZrP), and investigated it as an ion exchanger for heavy metals [85]. Specifically, the materials were synthesized via a modified mechanochemistry-based method, involving only grinding and heating. Typically, ZrOCl_2_·8H_2_O, Na_2_HPO_4_ and NaF (molar ratios 1:(2–4):0.3) were ground together in an agate mortar until a well-mixed paste was formed. Then, the mixture was heated to 120 °C for 24 h. For Na_2_HPO_4_:Zr > 2, the layered phase α-Zr(NaPO_4_)_2_·H_2_O with an interlayer distance of 8.59 Å was formed. For a Na_2_HPO_4_: Zr = 4, nanoplatelets with regular size and shape were obtained. In addition, nanorods were also observed, probably formed due to the presence of the fluoride ion. The performance of α-Na_2_ZrP as an ion exchanger was evaluated for several heavy metals (Pb, Cu, Zn, Co, Ni, Tl, Cd). It was found that the removal of Pb^2+^ was the highest, 99.9%, followed by Cu^2+^ (64.9%). In the absence of Pb^2+^, the removal efficiency for Cu^2+^ increased sharply to 98%. When Pb^2+^, Cu^2+^, Cd^2+^, and Tl^+^ were present together, the highest removal efficiency was still obtained for Pb^2+^. It is noteworthy that α-Na_2_ZrP was a much better ion-exchange material than the hydrogen form α-ZrP. Despite the high H^+^ concentration, the removal efficiency of Pb^2+^ and Cu^2+^ was ∼92% at pH 2 and increased to almost 100% at pH 3–5. The uptake of Tl^+^ was more susceptible to low pH, so that ∼98% removal efficiency was obtained at pH 3–5. Low uptakes of Tl^+^, Pb^2+^, and Cu^2+^ were observed only at pH 1. At pH 3, it is reasonable to suppose that part of the sodium ions in the solid was exchanged by H^+^, forming the monosodium-exchanged α-Zr(NaPO_4_)(HPO_4_)·5H_2_O. However, despite these phase changes with pH, the ion-exchange efficiency was not compromised due to the large interlayer spacing in α-Zr(NaPO_4_)(HPO_4_)·5H_2_O (*d* = 11.8 Å) or in the more hydrated θ-Zr(HPO_4_)_2_·6H_2_O (*d* = 10.4 Å). Competitive studies showed that, in the presence of various interferent ions, the removal efficiency for Pb^2+^, Cu^2+^, and Cd^2+^ was >90% in the presence of Na^+^, K^+^, and Mg^2+^, while the presence of an excess of Ca^2+^ resulted in reduced removal efficiencies for Pb^2+^, Cu^2+^, and Cd^2+^ by 10–30%.

**Figure 3:**
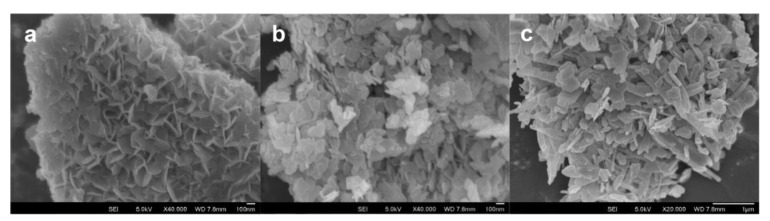
Scanning electron microscopy images of α-Zr-(NH_4_PO_4_)_2_·H_2_O prepared in the absence of HF (**a**), by using increasing amounts of HF (**b**,**c**) ([86], with permission of ACS).

Again, Chuah et al. synthesized α-Zr(NH_4_PO_4_)_2_·H_2_O by a single step minimalistic process, in which the reactants, ZrOCl_2_·8H_2_O and (NH_4_)_2_HPO_4_, were used in almost stoichiometric amounts in the solventless protocol where only the water of crystallization was present [86]. They found that by adding a small amount of NaF as a mineralizer, α-Zr(NH_4_PO_4_)_2_·H_2_O with good crystallinity and an interlayer distance of 9.44 Å was obtained. Scanning electron microscopy (SEM) images showed how the morphology of the α-Zr(NH_4_PO_4_)_2_·H_2_O particles depended on the amount of HF used for their preparation (Figure 3).

HF also affected the surface area of the samples. Specifically, it was found that the samples prepared by using HF had a surface area significantly lower than that of the compound synthesized without HF.

The sample prepared with the minimum amount of HF was used for sorption studies. The uptake of Pb^2+^ and Cu^2+^ was studied under acidic conditions. At pH 1, no sorption of Pb^2+^ and Cu^2+^ was observed, mainly for two reasons: first, at high H^+^ concentrations, there is the competition with heavy metal cations; secondly, at very low pH, ammonium ions are exchanged by protons forming α-Zr-(NH_4_PO_4_)_2_·H_2_O, which has an interlayer distance lower than that of the ammonium exchanged form (7.6 Å vs. 9.4 Å). At pH 2 and above, ≥98.9% Pb^2+^ was removed, while for Cu^2+^, the removal efficiency was close to 99.9% at pH ≥ 3. Sorption studies revealed a very strong affinity between the sorbent and the sorbate. Moreover, more than 60% of the target ions were removed from aqueous solutions in the first 10 min, and after 1 h the concentration became lower than 0.006 mmol/L. The high selectivity toward Pb^2+^ and Cu^2+^ was retained in the presence of other ions such as Na^+^, K^+^, Mg^2+^, Ba^2+^ and Ca^2+^ despite their much higher concentrations. Furthermore, the large difference in the uptake of Pb^2+^ and Cu^2+^ over that for Zn^2+^, Co^2+^ or Ni^2+^ also provides a method to separate these heavy metals.

Table 2 resumes the adsorption properties of some of the ZrP-based sorbent materials reported in the present review. The maximum ion uptake was referred to Pb^2+^, the most studied heavy metal, since it is the most common metal that the human body can absorb in toxic amounts. For batch experiments, the maximum Pb^2+^ uptake, expressed in meq per gram of sorbent, was reported, while the adsorption percentage was used for continuous adsorption mode experiments.

Table 2 shows that ZrP/melamine exhibited the highest metal uptake. This can be attributed to the complexing properties of N-rich molecules, similarly to crown ethers, as reported by Peng et al. [78]. Indeed, the metal uptake properties of ZrP intercalation compounds with nitrogen-containing molecules were already reported in 1985 by Ferragina et al., who studied the intercalation of 2,2’-bipyridyl into α-zirconium phosphate, and its coordination by Co^2+^, Ni^2+^, and Cu^2+^ [87].

Additionally, Takei et al. showed that γ-type zirconium phosphate intercalated with p-aminoazobenzene had an exceptional sorption ability towards rare earth elements, resulting about four times higher than that of unintercalated γ-ZrP [88]. They also proved that lanthanide uptake did not provoke the release of the azo-molecules.

## 4. Removal of Dyes

Organic dyes represent another important source of environmental contamination mainly coming from textile industries. Indeed, it was estimated that 1% to 15% of the dye is lost during dyeing processes and is released into wastewaters. Due to the large degree of aromaticity present in these molecules, dyes are difficult to degrade by traditional biological methods [89]. In the treatment of water contaminated by dyes, the adsorbent has a dual role: it should capture dyes from water and, at the same time, it should degrade them into environmentally compatible products, therefore also acting as a catalyst.

Organic dyes with positive charges or with protonable groups can be intercalated by zirconium phosphate through an ion-exchange process or an acid–base reaction involving POH groups [31].

In dye removal and degradation, zirconium phosphate is often employed as support for species with photocatalytic properties, with the aim to improve the surface area and, in turn, the number of available catalytic active sites.

Titania, silver nanoparticles, silver halides, graphene oxide, and carbon nitrides are some of the photocatalytic materials which were combined with zirconium phosphate [90]. The properties of the composites depend on the kind of photocatalyst and on its interaction with zirconium phosphate.

In 2007, Parida et al. tested the same material described in ref. [55], which was titania pillared zirconium phosphate, for the photocatalytic decolorisation of methylene blue (MB) [91]. The photo-oxidation of methylene blue was performed in batch by exposition to sunlight at room temperature. It was found that, with respect to pure ZrP, the presence of titania decreased the crystallite size which, in turn, increased the surface area and adsorption of methylene blue. This ultimately helped in increasing the initial rate of photodegradation/decolorisation of the dye.

Moreover, the photodecolorisation of methylene blue followed first-order kinetics, and at higher MB concentration the percentage of photodegradation decreased. On the other hand, with increasing the irradiation time, there was an increase in the percentage of photodecolorisation up to 240 min. It was also observed that the initial rate of photodegradation/decolorisation increased with increasing the H_2_O_2_ concentration, probably due to the formation of more OH^•^ radicals.

The effect of inorganic salts such as potassium persulphate and sodium chloride was also investigated. The effect of persulphate ion (electron scavenger) on the photocatalytic degradation of MB resulted in an increase in the initial rate of photodegradation of methylene blue with increasing the persulphate amount, attaining a 100% degradation in 4 h. Differently, with increasing the concentration of sodium chloride, the initial rate of photodegradation of MB decreased due to the hole scavenging properties of Cl^−^ ion.

The efficiency of titania pillared zirconium phosphate was comparable with that of other catalysts reported in the literature.

It is well known that noble metallic nanoparticles, such as gold and silver, exhibit unique spectral properties due to their surface plasmon resonance properties, which made them promising materials for several applications, such as photocatalysis, due to their ability to absorb visible light [92,93]. Moreover, the combination of silver nanoparticles with silver halides produces heterojunctions, in which the two components act so as to polarize the photo-induced charges, facilitating electron-hole separation, and in turn, the photocatalytic efficiency of the material [94].

Saffaj and coworkers studied the effect of silver loaded zirconium phosphate on the photodegradation of methylene blue [95]. Crystalline α-Zirconium phosphate was prepared by refluxing amorphous zirconium phosphate in a solution of phosphoric acid. For silver activation, amorphous α-ZrP was added to a solution of silver nitrate. Then, phosphoric acid was added and the mixture was refluxed for 2 days to yield a precipitate denoted Ag–ZrP. Both α-ZrP and Ag–ZrP exhibited an interlayer distance of 7.6 Å, but α -ZrP had a higher degree of crystallinity than Ag–ZrP. However, regarding the oxidation state of silver, the authors did not clarify if it is present as ion or metallic silver.

Methylene blue was selected as a model for the photocatalytic degradation experiments because it is a non-volatile and common contaminant in industrial wastewaters. A low-pressure mercury lamp UV was used as an artificial sunlight source. It was found that the photocatalytic efficiency of the dye degradation over Ag–ZrP and α-ZrP was highly pH dependent. Specifically, the photocatalytic efficiency increased with increasing pH values. In strong acidic conditions, the decolorization of the dye was negligible, while it dramatically increased at pH 8. Both α-ZrP and Ag–ZrP surfaces were positively charged below pH 4. Methylene blue is a cationic dye in aqueous solution and it can keep its cationic configuration in the pH range 3–11. At basic pH, electrostatic interactions between the negative catalyst surface and cationic dye led to strong adsorption of the latter on the inorganic support (despite the higher risk of hydrolysis of ZrP, occurring in basic conditions, note of the author).

Moreover, Ag–ZrP exhibited a higher degree of photocatalytic efficiency compared to α-ZrP. Specifically, a decrease in the concentration of MB in the solution around 90% was achieved with Ag–ZrP within 70 min, while it was less than 60% with α-ZrP in the same time range. Enhanced degradation with Ag–ZrP under UV irradiation was ascribed to the effects of Ag ions acting as electron traps leading to better electron excitation. 

Composites based on zirconium phosphate and silver/silver halide heterojunctions demonstrated efficiency in the photodegradation of Rhodamine B (RhB) [96,97]. They were quickly and easily obtained by using nanosized silver(I) exchanged α-zirconium phosphate as a precipitating agent for silver halides. Specifically, a nanosized zirconium phosphate, prepared as reported by Pica et al. [55], first was reacted with different amounts of silver(I) acetate, then treated with HCl or HBr solutions in order to promote the precpitation of AgCl or AgBr. All composites contained AgX particles with size ≤1 μm, and UV-Vis diffuse reflectance spectroscopy proved that ZP/AgX was effectively converted into ZP/x(Ag@AgX) after a few minutes of irradiation with a halogen lamp.

The photodegradation of RhB was studied in batch and the photocatalytic properties of the composites were compared with those of a pure AgX sample with particle size in the range of 0.5–2 μm. It was observed that, during irradiation, the absorbance decreased and the wavelength of the absorption maximum (λ_m_) slowly shifted toward lower λ values. Watanabe et al., in 1977, proved that the ipsochromic shift of λ_m_ was due to de-ethylation occurring in a stepwise manner, and to cleavage processes [98]. It was found that, both for AgX and ZP/AgX or ZrP/AgCl, the degradation process started with RhB de-ethylation, followed by the cleavage of the de-ethylated species. However, the composites with 53 and 58 wt% of AgBr and AgCl, respectively, turned out to have better catalytic properties than the corresponding pure AgX samples. Specifically, the percentage of chromophore cleavage in the presence of ZP/AgBr and ZP/AgCl was around 90% after 6 min and 10 min, respectively, while in the presence of pure AgX, a 90% of percentage cleavage was achieved after 30 min. Moreover, reusage tests of the composite photocatalysts proved that they are stable for at least three cycles.

Ye et al. fabricated α-zirconium phosphate-pillared reduced graphene oxide (rGO–ZrP) composites for the removal of MB from aqueous solutions [99]. GO was synthesized from natural graphite powder by a modified Hummers method, while ZrP was synthesized by refluxing amorphous ZrP for 7 days in the presence of phosphoric acid. The GO–ZrP composite was prepared from a ZrP/methylamine intercalation compound, to which GO was slowly added. Then, hydrazine was added in order to reduce GO. BET specific surface areas of rGO–ZrP and rGO were 573 and 312 m^2^ g^−1^, respectively. It is interesting that, with increasing the storage time in water, the BET specific surface area of rGO decreased to 44 m^2^ g^−1^, while that of rGO–ZrP only decreased to 523 m^2^ g^−1^, suggesting that the presence of ZrP hampered the rGO reaggregation to some extent.

The adsorption tests of MB onto the rGO–ZrP were conducted in a batch experiment at 30 °C. The maximum adsorption capacitiy of MB onto rGO–ZrP was about 90% higher than that onto rGO. With increasing the storage days in water, the maximum adsorption capacity of MB onto the rGO dramatically decreased, while that onto rGO–ZrP slightly decreased. Moreover, the maximum adsorption capacity of rGO–ZrP almost remained constant during the first six cycles of the adsorption–desorption process.

Chen et al. recently prepared α-zirconium phosphate/carbon nitride composites (ZrP/C_3_N_4_), with different ZrP/C_3_N_4_ mass ratios, and investigated their photocatalytic activity toward Rhodamine B under UV irradiation [100]. Zirconium phosphate was prepared by the hydrothermal reaction of zirconyl chloride and phosphoric acid, while carbon nitride was prepared by the calcination of urea. ZrP/C_3_N_4_ composites were prepared by mixing and sonicating different amounts of the two components in water. It was found that the particle size of C_3_N_4_ was in the range 200 nm–a few micrometers and higher amounts of C_3_N_4_ restricted the re-stacking process of α-ZrP nanoparticles. Photoluminescence spectra showed that when α-ZrP nanoparticles were composited with C_3_N_4_ nanosheets, a heterostructure was formed, and the high recombination rate of photogenerated carriers, which was the intrinsic property of C_3_N_4_, was efficiently reduced. Photocatalytic activity was tested under irradiation with UV-Vis light.

The photodegradation efficiency of RhB by ZrP under visible light was negligible since it cannot be excited by visible light irradiation. After incorporation with C_3_N_4_, the efficiency was improved slightly. In the condition of ultraviolet light irradiation, the photodegradation efficiency of ZrP/C_3_N_4_ composite material (ZrP:C_3_N_4_ mass ratio = 1:1) was much higher than that of α-ZrP and C_3_N_4_. After irradiation for 10 min, the photodegradation efficiency of α-ZrP and C_3_N_4_ was 64 and 67%, respectively, while the photodegradation efficiency of ZrP/C_3_N_4_ (ZrP:C_3_N_4_ mass ratio = 1:1) reached 98% in the same time range. The degradation efficiency of all composite systems reached above 90% after 18 min. Among those, the composite with ZrP: C_3_N_4_ mass ratio 1:2 had the highest degradation efficiency (≈100%).

The authors inferred that under UV irradiation, the photoinduced holes on the valence band of α-ZrP were transferred to the valence band of C_3_N_4_ through the heterostructure. The photogenerated electrons from the conduction band of C_3_N_4_ were transferred to the conduction band of α-ZrP, which achieved effective separation of photogenerated electron-hole pairs and reduced the electron-hole recombination. Moreover, an appropriate amount of C_3_N_4_ would benefit the separation of photogenerated electron-hole pairs, while an excess of C_3_N_4_ would block, to some extent, the ultraviolet light arriving at the surface of ZrP nanoparticles and then cause a decrease in the photodegradation rate.

Naushad et al. proposed a gelatin-Zr(IV) phosphate nanocomposite to remove toxic dyes from water [101]. Gelatin is a naturally occurring macromolecular and biodegradable protein produced by partial hydrolysis of collagen synthesized from the skins, white connective tissues and bones of animals.

**Figure 4:**
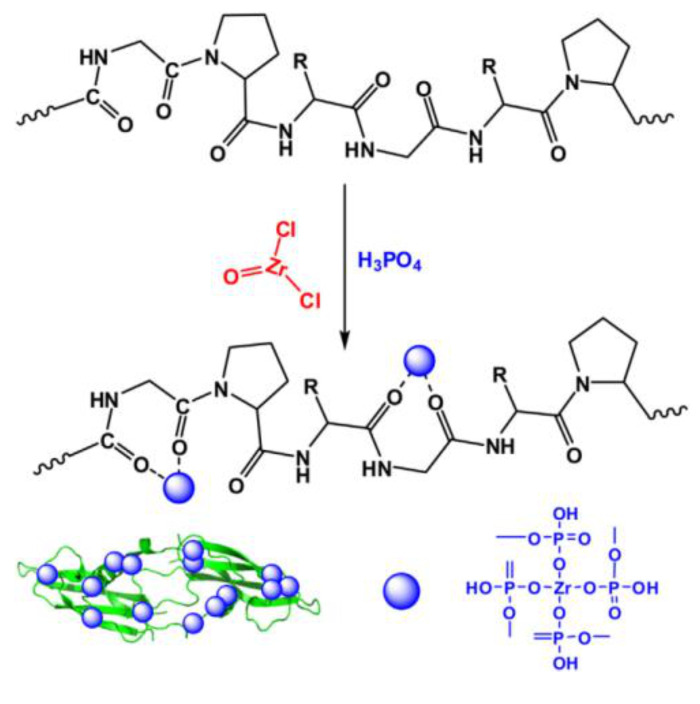
Proposed mechanism for the synthesis of gelatin-Zr(IV) phosphate nanocomposite (GT/ZPNC) ([101], with permission of Elsevier).

Gelatin-Zr(IV) phosphate nanocomposite (GT/ZPNC) was synthesized using the sol–gel method. Specifically, ZrOCl_2_·8H_2_O and H_3_PO_4_ were mixed with continuous stirring at room temperature and the pH was kept between 0 and 1. Gelatin was then added in hot water to prepare gelatin gels, which were added to precipitates of Zr(IV) phosphate under continuous stirring (Figure 4).

For the sake of clarity, the structure of ZrOCl_2_ reported in Figure 4 is quite unusual and unprobable, since, as reported by Clearfield et al., zirconyl chloride does not contain ZrO^2+^ species but a tetrameric species [Zr(OH)_2_·4H_2_O]_4_^8+^, which also exists in acidic solution of ZrOCl_2_·8H_2_O. Chlorine is not bonded to Zr but it is present as a Cl^−^ ion [102].

The photocatalytic behaviour of GT/ZPNC particles was investigated against MB and FG (Fast green) dyes under solar light using batch process. The removal of FG dye in visible light reached about 90% within five hours, while in the dark it reached only 44%. Similar results were obtained for MB: about 38% of dye was removed in the dark and the removal increased up to 88% within five hours when exposed to sunlight. However, the authors did not carry out experiments in order to clarify if the higher dye removal in the presence of GT/ZPNC was due to a simple adsorption or to a photodegradation process.

## 5. Conclusions

Pollutant removal from water through adsorption is considered one of the most effective and competitive processes. The optimal adsorbents should be cheap, chemically stable, have a high surface area in order to make available a large number of active sites, and be easily regenerated and recoverable.

Among unconventional sorbents, layered zirconium phosphate has been investigated, both in batch and continuous conditions, as a potential material for the removal of heavy metals and dye due to its good chemical and thermal stability and, above all, its excellent cation exchange and intercalation properties.

Both amorphous and crystalline ZrP materials have been investigated as sorbent materials, often in the form of composites, in combination with organic polymers or other inorganic compounds, in order to have feasible hybrid sorbents for continuous and large-scale applications. Neutral and charged polymers, as well as inorganic compounds such as titania, graphene oxide, crown ethers, and N-rich molecules, have been used for the preparation of ZrP composites, which proved to be suitable and promising for a continuous adsorption mode. Among ZrP composites, those with N-rich molecules exhibited the highest metal uptake due to the chelating properties of these molecules toward metals.

Heavy metal uptake, occurring through ion exchange processes, has been studied as a function of different experimental conditions (pH, concentration of the sorbent and the sorbate, temperature, etc.), proving that the uptake is more efficient in moderate acidic conditions. The use of ZrP composites, which enable the achievement of higher surface areas, is beneficial for their adsorbent properties. Regardless of the characteristics of the sorbent, the highest affinity is observed toward lead and copper. Moreover, the affinity of zirconium phosphate-based materials for heavy metals is generally significantly higher than for alkaline and alkaline earth metals.

The uptake of organic dyes by ZrP can occur through both acid–base interactions and ion exchange processes. It is desirable that dyes should not be simply adsorbed on the solid material, but also degraded to nontoxic products, and photocatalytic degradation is often used. ZrP combined with photosensitive materials, among them titania, graphene oxide, carbon nitrides, silver nanoparticles and silver/silver halide heterojunctions, exhibits enhanced photocatalytic efficiency due to a more efficient light absorption and electron-hole separation.

The major part of the reported studies refer to lab scale experiments, proving that the use of zirconium phosphate as a sorbent material is still in the phase of fundametal research, and further experiments are undoubtedly necessary to prove the effective possibility to apply these materials on a large scale, where other aspects, such as economic advantages or disadvantages, should be evaluated.

Further developments of this topic are currently under investigation in my research group. New materials, based on layered zirconium phosphonates, bearing carboxylic and amino groups, covalently bonded to the inorganic layer, are promising potential sorbents for heavy metals and dyes. The idea was born from the synthesis of a novel mixed zirconium phosphate/phosphonate based on glyphosine, specifically a zirconium phosphate glycine-N, Nbismethylphosphonate, in which the presence of the carboxyl and amino groups, due to their coordinative capabilities, enhances the affinity of this material towards transition metals [103,104,105] and, at the same time, the presence of acidic groups could promote the adsorption of basic dyes.

## Figures and Tables

**Table 1:** Upper limit concentrations, according WHO in drinking water for some heavy metals and their harmful effects on the human body [4].

Heavy Metal	Upper Limit Concentration in Drinking Water (mg/L)	Toxic Effects on Human Body
Lead	0.01	Harmful to heart, bones, intestines, reproductive and nervous systems.
Copper	2	Mucosal irritation, capillary damage, hepatic and renal damage, central nervous system damage.
Cadmium	0.003	Bone lesions, cancer, lung insufficiency, hypertension.
Zinc	3	Gastrointestinal effects.
Nickel	0.02	Cancer, skin allergy, lung fibrosis.
Mercury	0.001	Kidney disease, haemorrhagic gastritis and colitis, brain damage, cancer.
Chromium	0.05 (hexavalent chromium)	Cancer, healing ulcers.

**Table 2:** Maximum Pb^2+^ uptake, or adsorption percentage values, for ZrP-based sorbent materials.

Sorbent	Maximum Pb^2+^ Uptake (meq/g) or Adsorption Percentage (%)	Adsorption Mode	Ref.
Amorphous ZrP	≈3 at pH 5.5	Batch	[42]
ZrP monoliths	100%	Continuous	[82]
Zr(NaPO_4_)_2_⋅H_2_O	≈5 at pH 5	Batch	[85]
Zr(NH_4_PO_4_)_2_⋅H_2_O	≈3.8 at pH ≈4.3–5	Batch	[86]
Amorphous ZrP/D001	100%	Continuous	[59]
α, γ-ZrP/mesoporous polystyrene	≈1.6 at pH = 5	Batch	[60]
Amorphous ZrP/polysulfone capsules	≈3 at pH 5.75	Batch	[62]
α-ZrP/polyaniline	≈1	Electrochemical quartz crystal microbalance	[68]
Amorphous ZrP/ (polyvinyl alcohol-polyvinylidene fluoride)	≈1.2 at pH 5.5	Batch	[70]
ZrP nanoparticles/cellulose	60%	Continuous	[71]
Amorphous ZrP/graphene oxide	≈3.5 at pH = 6	Batch	[74]
Amorphous ZrP/melamine	≈9.7 at pH 5.5	Batch	[81]
